# Modulation of Higher Order Chromatin Conformation in Mammalian Cell Nuclei Can Be Mediated by Polyamines and Divalent Cations

**DOI:** 10.1371/journal.pone.0067689

**Published:** 2013-06-26

**Authors:** Ashwat Visvanathan, Kashif Ahmed, Liron Even-Faitelson, David Lleres, David P. Bazett-Jones, Angus I. Lamond

**Affiliations:** 1 Centre for Gene Regulation and Expression, College of Life Sciences, University of Dundee, Dundee, United Kingdom; 2 Genetics and Genome Biology Program, The Hospital for Sick Children, Toronto, Canada; University of Quebect at Trois-Rivieres, Canada

## Abstract

The organisation of the large volume of mammalian genomic DNA within cell nuclei requires mechanisms to regulate chromatin compaction involving the reversible formation of higher order structures. The compaction state of chromatin varies between interphase and mitosis and is also subject to rapid and reversible change upon ATP depletion/repletion. In this study we have investigated mechanisms that may be involved in promoting the hyper-condensation of chromatin when ATP levels are depleted by treating cells with sodium azide and 2-deoxyglucose. Chromatin conformation was analysed in both live and permeabilised HeLa cells using FLIM-FRET, high resolution fluorescence microscopy and by electron spectroscopic imaging microscopy. We show that chromatin compaction following ATP depletion is not caused by loss of transcription activity and that it can occur at a similar level in both interphase and mitotic cells. Analysis of both live and permeabilised HeLa cells shows that chromatin conformation within nuclei is strongly influenced by the levels of divalent cations, including calcium and magnesium. While ATP depletion results in an increase in the level of unbound calcium, chromatin condensation still occurs even in the presence of a calcium chelator. Chromatin compaction is shown to be strongly affected by small changes in the levels of polyamines, including spermine and spermidine. The data are consistent with a model in which the increased intracellular pool of polyamines and divalent cations, resulting from depletion of ATP, bind to DNA and contribute to the large scale hyper-compaction of chromatin by a charge neutralisation mechanism.

## Introduction

All known eukaryotes store their DNA inside nuclei in the form of chromatin, wherein the DNA associates with histones to form nucleosomes. A major function of chromatin is in organization and compaction of long genomic DNA within the confined space of the nucleus. The basic unit of chromatin, the nucleosome, consists of an octamer of core histones with 147 bp of DNA wrapped around in 1.7 left handed superhelical turns [1]. The nucleosomes are separated via a ∼10–80 bp segment of linker DNA that is bound by histone H1. The nucleosome octamer is formed from two copies of each of the histones H2A, H2B, H3 and H4. The core histones have many positively charged lysine and arginine residues, which can neutralize ∼60% of the negative charge in the polyphosphate DNA backbone [2]. The nucleosomes are not static complexes and can either dissociate, or move/slide along the DNA [3], [4]. The formation of poly-nucleosome chromatin through association of DNA with the histone octamer results in a ∼5–10 fold compaction of DNA [5]. This poly-nucleosome ‘beads on a string’ arrangement corresponds to the ‘10 nm’ form of chromatin. However, the remaining ∼40% of the negative charge in the polyphosphate DNA backbone can still cause repulsion and thus act as a barrier to further chromatin compaction.

Under cell-free, *in vitro* conditions, the 10 nm form of chromatin can be further condensed to form a more compact fibre that is approximately 30 nm in width, resulting in an overall compaction factor of about 50 fold. This 30 nm fibre can be formed *in vitro* by further charge neutralisation of the sugar-phosphate backbone through the addition of polyvalent cations. The 30 nm structure is one of the most well studied forms of chromatin *in vitro* [4]. However, it should be noted that so far the 30 nm structure has not been observed directly *in vivo* in intact mammalian cells [6], [7], [8].

The charge state of chromatin is viewed as an important determinant of compaction and this can also be modulated via post translational modifications (PTMs) to histones. Histone PTMs can either add, or remove, charge and also can form binding sites for proteins [9]. Reversible changes in the modification states of histones have been shown to affect the structure of chromatin [10]. Although histone modifications can recruit other proteins to bind chromatin, histone PTM-induced changes in chromatin structure have been seen also in cell free, *in vitro* systems, indicating that charge altering modifications can, potentially, influence chromatin structure directly, independent of other factors [10].

Detailed information on the modulation of chromatin structure has come mostly from *in vitro* experiments and particularly from experiments using reconstituted chromatin. However, the nuclear chromatin environment is more complex and contains many protein, RNA and other components that are not present in reconstituted systems and that have the potential to influence chromatin structure and compaction. Hence, it is possible that chromatin may behave differently *in vivo* from the properties observed using reconstituted systems. This may explain why the 30 nm fibre, which has been very well documented *in vitro*, has not been observed even in the most compact forms of mammalian chromatin *in vivo* [8].

In this study we use a FLIM-FRET (Fluorescence Lifetime Imaging Microscopy-Förster Resonance Energy Transfer) based method to assess changes in chromatin conformation *in vivo* in live cells [11]. In this system we analyse cells stably co-expressing forms of histone H2B fused to either EGFP or mCherry. The relative proximity of nucleosomes is quantified by measuring the fluorescence lifetime of H2B-EGFP in the presence of mCherry-H2B, which can form a FRET pair when they are in close proximity. Compaction of chromatin will increase the number of EGFP-H2B molecules that come into close proximity with both mCherry-H2B and other chromatin components. Both the resulting FRET [12] and collisional quenching [13] from the molecular crowding, can lead to a decrease in the fluorescence lifetime of EGFP. The decrease in the lifetime of EGFP thus provides a read out for the change in chromatin conformation that can be applied to intact, live cells.

Here we have used *in vivo* FLIM-FRET to probe how chromatin structure responds to changes in the concentration of polyvalent cations and to ATP levels within the environment of cell nuclei. The resulting data show that changes in polycation levels can cause major transitions in DNA compaction within nuclei and suggest that this may also account, at least in part, for the changes in DNA compaction that accompany depletion of ATP.

## Results

### ATP Depletion Leads to a Global Increase in Chromatin Compaction

**Figure 1:**
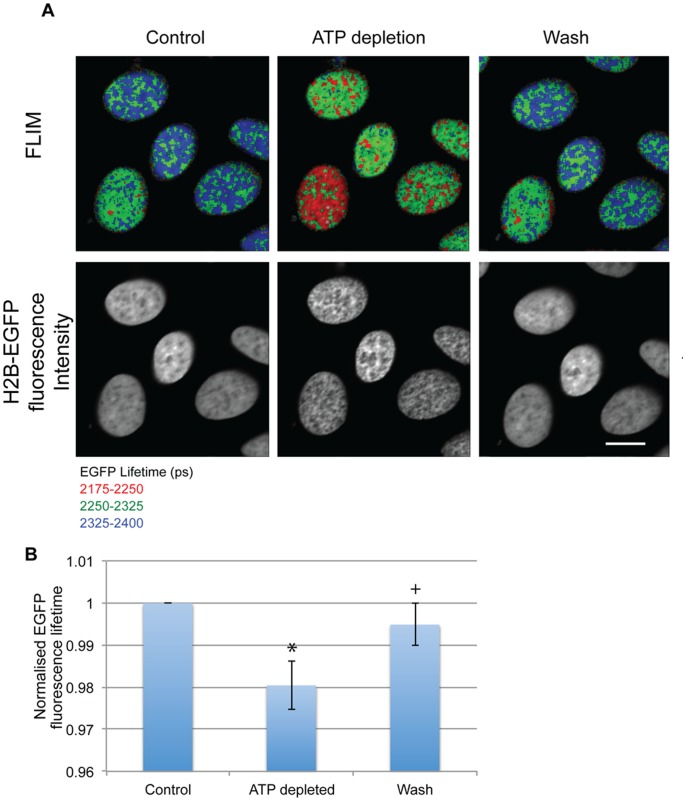
ATP depletion leads to increased chromatin compaction. (A) FLIM images show the reduction of fluorescence lifetime in Hela^H2B-2FP^ cells upon ATP depletion and an increase when the drugs are washed away. The increase in compaction is also seen in the intensity images (Scale bar –10 µm). (B) The chart shows the average decrease and subsequent increase in EGFP fluorescence lifetime values normalised to the lifetime of the control nuclei. Error bars indicate standard deviation for values collected for 13 cells from 3 independent experiments. (* p-value = 3.7×10^−8^,+p-value = 0.0046).

Depletion of ATP *in vivo* can be accomplished with the addition of drugs that inhibit both oxidative phosphorylation as well as glycolytic pathways simultaneously [14]. It has been shown that depletion of ATP causes major changes in the nucleus, including the formation of electron-dense barriers inside the nucleoplasm [11], [15], which correspond to compacted chromatin. HeLa^H2B-2FP^ cells stably expressing H2B-EGFP and mCherry-H2B were depleted of ATP by treating for 10 minutes with 10 mM sodium azide and 50 mM 2-deoxy glucose. Cells were imaged by 2 photon fluorescence microscopy and TCSPC-FLIM (Time Correlated Single Photon Counting – Förster Resonance Energy Transfer) measurements acquired before and after addition of the drugs. The sodium azide and 2-deoxyglucose were then washed away and another set of FLIM measurements were acquired for the same set of cells, 10 minutes after washing the cells with fresh medium (Fig. 1).

As judged by both the change in EGFP fluorescence lifetime and the appearance of the chromatin in the fluorescence intensity images, there was a drastic increase in the compaction of chromatin upon addition of the sodium azide and 2-deoxyglucose (Fig. 1). The compaction effect is rapid and reversible. Compaction is seen within 10 minutes of adding the sodium azide and 2-deoxyglucose and is reversed within 10 minutes of their removal, with the EGFP fluorescence lifetime reverting closer to the control levels (Fig. 1). These data are consistent with previous observations [11], confirming that chromatin in interphase cells compacts reversibly with the depletion/repletion of ATP.

### ATP-dependent Chromatin Compaction is not Caused by Transcription Inhibition

Actively transcribed regions have been shown to be associated with decompacted regions of chromatin in interphase cells [16]. Depletion of ATP will lead to the inhibition of transcription [11]. To test if the compaction of interphase chromatin associated with depletion of ATP is brought about primarily by the inhibition of transcription, transcription was blocked with inhibitors in the absence of ATP depletion and the resulting effect on chromatin compaction studied.

**Figure 2:**
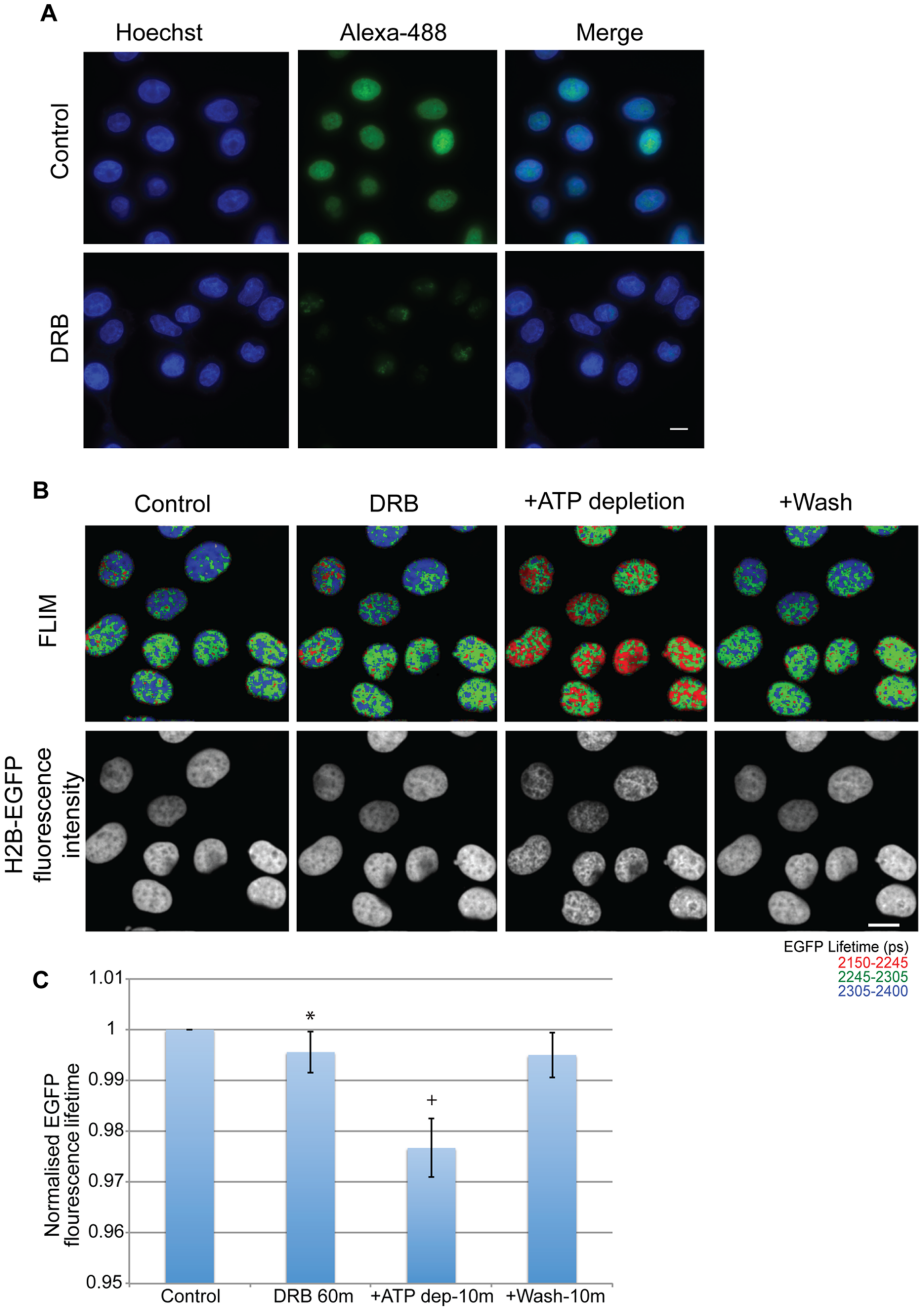
Transcription inhibition does not lead to a drastic increase in chromatin compaction. (A) the panel shows HeLa cells with nuclear (Hoechst) and nascent RNA (Alexa-488) staining (pulse labeled with EU for 30 m). The upper panel shows control cells and the lower panel shows cells treated with DRB at a concentration of 25 µg/ml for 60 minutes. (Scale bar-10 µm) (B) FLIM images showing a decrease in fluorescence lifetime upon ATP depletion for 10 minutes in cells treated with DRB at a concentration of 25 µg/ml for 60 minutes, which is reversed upon washing away of the drugs. The resulting chromatin compaction is evident from the Intensity images of H2B-GFP fluorescence. (Scale bar-10 µm) (C) The chart shows the averaged normalised fluorescence lifetime values of each of the cells upon treatment. Error bars indicate standard deviation for values from 18 nuclei from 3 replicates. (* p-value = 0.0002,+p-value = 3.7×10^−12^).

Transcription was inhibited by the addition of 5,6-dichlorobenzimidazole (DRB) at a concentration of 25 µg/ml [17]. DRB is an inhibitor of Cdk-activating kinase, and consequently prevents elongation by RNA polymerase II [18]. To assess the extent of transcription *in vivo*, cells were pulse labeled with 5-ethynyl uridine for 30 minutes prior to fixation. The newly synthesised RNA incorporates 5-ethynyl uridine, which can then be visualised by conjugating it with the Alexa Fluor-488 fluorophore. At 60 minutes after addition of DRB, transcription in the nucleoplasm was inhibited, as seen from the reduction in Alexa Fluor-488 fluorescence compared with the control untreated cells (Fig. 2A).

To determine if the inhibition of transcription led to changes in chromatin compaction, FLIM measurements were acquired from HeLa^H2B-2FP^ cells both before, and after, treatment with 25 µg/ml DRB for 60 minutes. The measured EGFP fluorescence lifetime values showed a small decrease after treatment with DRB. Visual inspection of the fluorescence images of DRB-treated cells also does not show the obvious change in compaction observed after ATP depletion. Subsequently treating the DRB-inhibited cells with sodium azide and 2-deoxyglucose to deplete ATP caused an increase in compaction, as judged from the H2B-EGFP fluorescence intensity images (Fig. 2B). There was also a drastic decrease in the fluorescence lifetime of EGFP in the cells after ATP depletion (Fig. 2). While DRB inhibits RNA polymerase II but not RNA polymerase I, we nonetheless see no differential effect on chromatin compaction in nucleoli as opposed to the nucleoplasm after DRB treatment, which supports the view that the extent of chromatin compaction after ATP depletion does not correlate directly with transcription activity. Furthermore, while DRB blocks RNA polymerase II activity by inhibition of Cdk-activating kinase, we note that treatment of cells with the broad spectrum kinase inhibitor staurosporine also did not prevent compaction of chromatin after ATP depletion (data not shown). This argues against a major role for general kinase activity in promoting the ATP depletion-dependent compaction.

Next, to examine the potential involvement of transcription further, we examined whether ATP depletion also affected chromosome conformation in cells undergoing mitosis, where transcription is absent [19].

### ATP Depletion Increases Compaction of Mitotic Chromosomes

**Figure 3:**
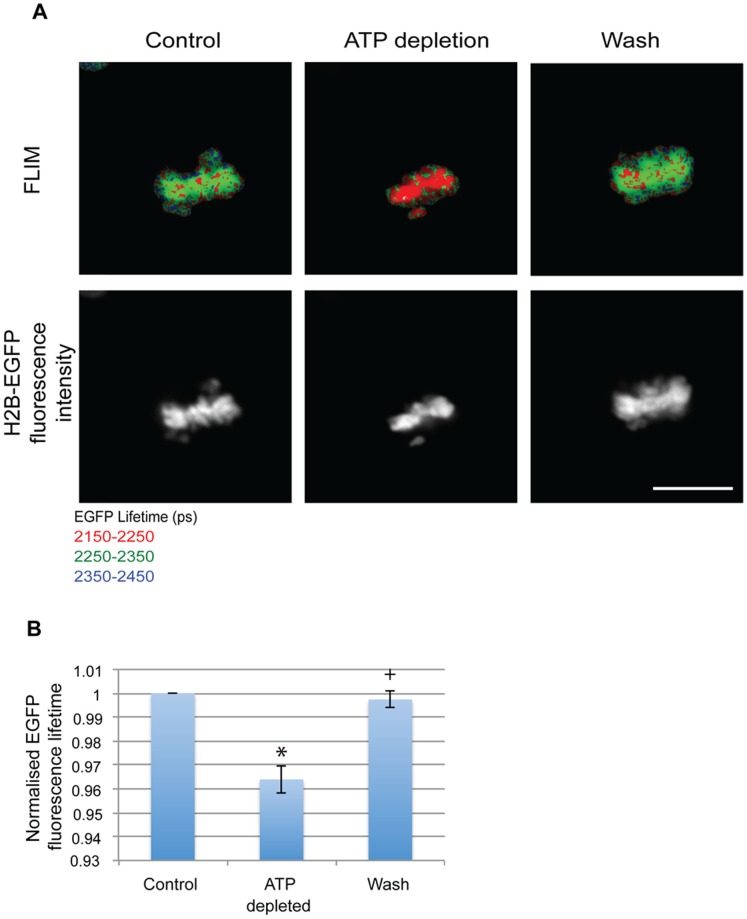
Mitotic chromatin compacts with ATP depletion. (A) FLIM images showing a decrease in EGFP fluorescence lifetime on ATP depletion and its subsequent increase to control levels when the drugs are washed away in Hela^H2B-2FP^ cells (Scale bar - 10 µm). (B) Chart showing the average EGFP fluorescence lifetime normalised to control values from 9 mitotic cells acquired from 7 individual experiments showing changes upon ATP depletion and repletion. Error bars indicate standard deviation. (* p-value = 3.7×10^−6^,+p-value = 0.144).

Mitotic chromosomes are formed by condensation of the less compacted interphase chromosomes. To check if the compaction of chromatin upon ATP depletion is either specific for interphase chromatin, or if the effect can also be seen in the already highly compacted mitotic chromosomes, FLIM measurements of mitotic chromosomes before and after ATP depletion were acquired as described in previous experiments. ATP was depleted by addition of sodium azide and 2-deoxyglucose to HeLa^H2B-2FP^ cells for 10 minutes. Mitotic cells were visually selected by fluorescence of the tagged histones showing aligned condensed chromosomes, characteristic of the metaphase stage of mitosis. ATP depletion resulted in a decrease in the measured fluorescence lifetime of EGFP in the mitotic cells, indicating a structural transition and further compaction of the mitotic chromosomes (Fig. 3). As with interphase cells, this compaction could be reversed upon repletion of ATP by washing away the sodium azide and 2-deoxyglucose. This result was rather surprising, because the data indicate that despite the more highly condensed state of mitotic chromosomes they are still able to undergo a similar reversible hyper-compaction upon ATP depletion to that seen for interphase chromosomes. We therefore infer that the transition in chromatin structure triggered by ATP depletion is distinct from the structural transition that compacts chromosomes in mitotic cells. Furthermore, the data provide additional evidence that the ATP-dependent compaction of chromatin is unlikely to arise as an indirect effect of blocking transcription because the mitotic chromosomes are not active in either transcription, or DNA replication [19]. We conclude from these data that the change in chromatin compaction caused by ATP depletion cannot result simply from the loss of transcription.

We next examined how ATP depletion can affect the intracellular levels of unbound cations, which can potentially influence chromatin compaction levels.

### ATP Depletion Leads to an Increase in the Intracellular Ca^2+^ Concentration

*In vitro*, nucleosome arrays compact with an increase in Ca^2+^ concentration [20]. In addition, Ca^2+^ has also been implicated in compaction of mitotic chromosomes, as shown by ion microscopy, wherein there is an increased concentration of Ca^2+^ on mitotic chromosomes when compared with interphase chromatin [2]. However, there have been conflicting reports on the differential regulation of Ca^2+^ concentrations between the nucleus and cytoplasm in interphase cells [21]. Intracellular Ca^2+^ has been shown to increase after ATP depletion [22].

To examine whether there was an increase in calcium levels after ATP depletion in the HeLa^H2B-2FP^ cells, calcium was imaged using the cell permeable dye Fluo4-AM. On entering the cell, Fluo4-AM is cleaved into the active form by cellular esterases, thereby rendering it unable to permeate cellular membranes and hence trapping it within cells. When active, Fluo4 fluoresces only when bound to calcium [23].

**Figure 4:**
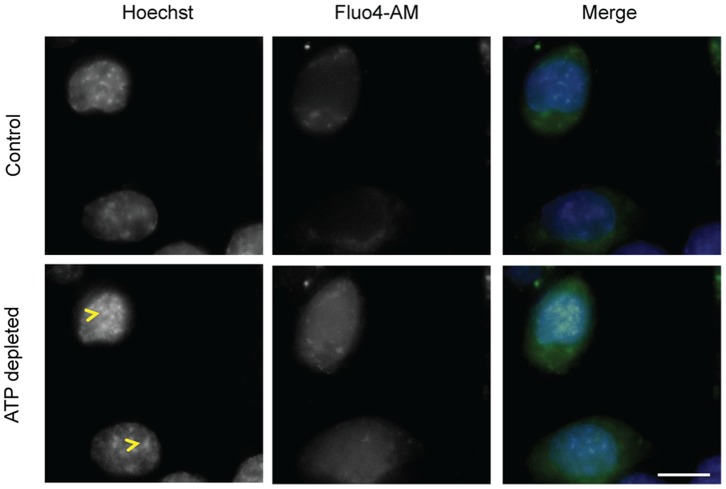
ATP depletion leads to an increase in intracellular free calcium levels. Fluo4-AM loaded HeLa cells were imaged before and after ATP depletion. The increase in fluorescence indicates an increase in free intracellular calcium levels (p-value = 0.0001). The increase in chromatin compaction (yellow arrow head) can be seen from Hoechst staining after ATP depletion. (Scale bar –10 µm).

HeLa cells loaded with Fluo4-AM were imaged both before and after ATP depletion. Control cells showed lower fluorescence in the nuclear regions, as identified by nuclear staining with Hoechst 33342, when compared with cytoplasmic regions (Fig. 4). This indicates that, as expected, in interphase cells there are lower calcium levels within the nucleus than in the cytoplasm. Upon ATP depletion, there was a significant increase in Fluo4 fluorescence (p value- 0.006), showing an increase in free Ca^2+^ concentration, which was homogenously distributed throughout the cell (Fig. 4). The concomitant increased compaction of chromatin can also be seen with the parallel Hoechst 33342 staining (Fig. 4, arrowheads). Next, therefore, we examined how the concentration of divalent cations might affect the level of chromatin compaction in nuclei, using a permeabilised cell system.

### Chromatin Compaction in Permeabilised Cells Increases with Increasing Divalent Cation Concentration

Ca^2+^ and Mg^2+^ are the two most abundant divalent cations present in cells and function as cofactors for multiple enzymes. Increasing the concentration of either Mg^2+^ or Ca^2+^ has been shown to increase compaction of nucleosome arrays *in vitro* [20]. Increasing Mg^2+^ concentration increases the association between nucleosomes, both within the same nucleosome array and also with nucleosomes of different arrays [24].

**Figure 5:**
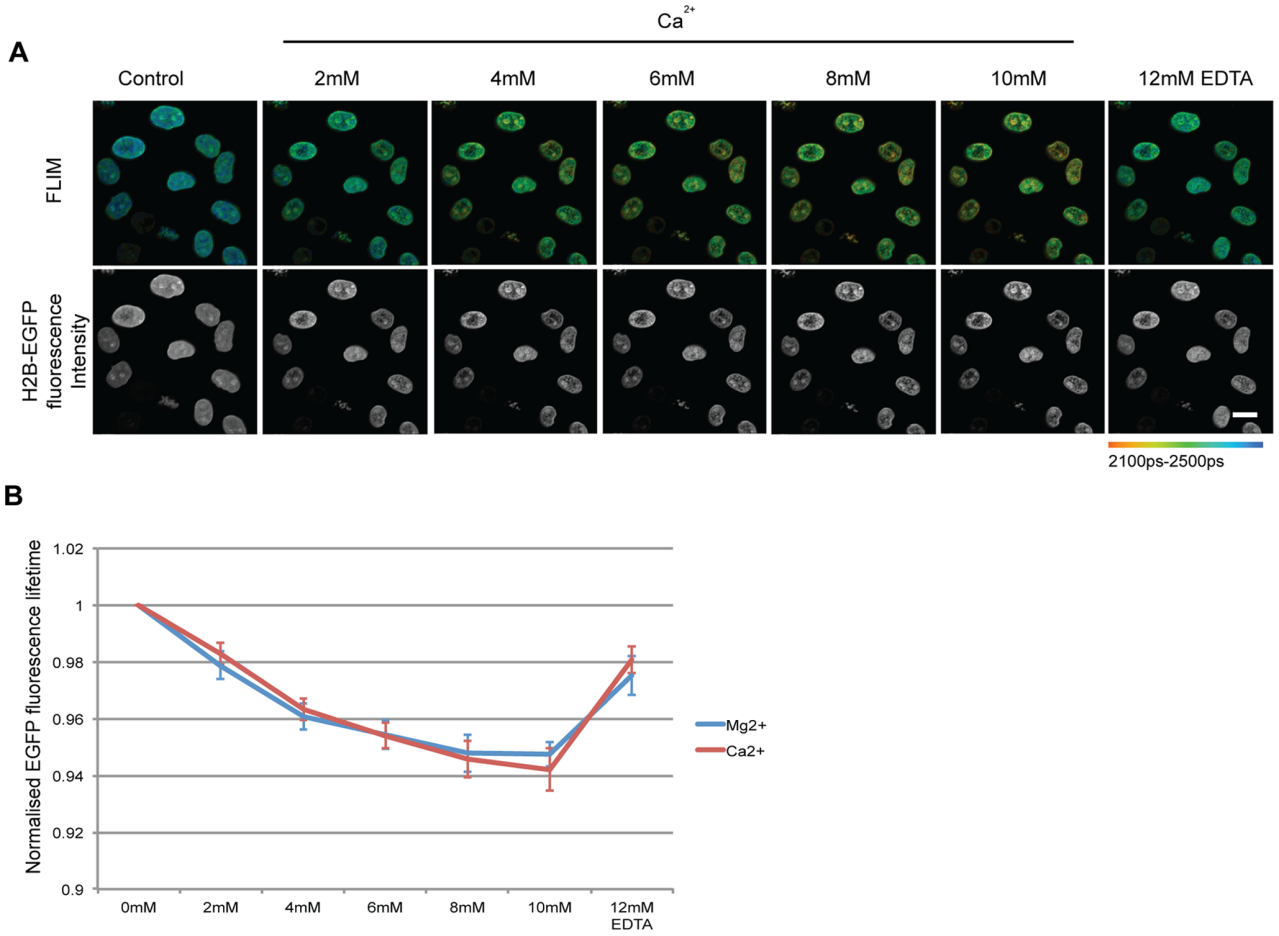
Increasing divalent cation concentration leads to an increase in chromatin compaction in permeabilised cells. (A) FLIM images of permeabilised Hela^H2B-2FP^ cells with increasing Ca^2+^ concentration. There is a decrease in lifetime with increasing Ca^2+^ concentrations and an increase with the addition of 12 mM of EDTA. The increase in compaction is also seen with the fluorescence intensity image. (Scale bar –10 µm). (B) Average EGFP fluorescence lifetime normalised to the value of control cells are plotted against the varying concentration of Ca^2+^ and Mg^2+^. Error bars indicate standard deviation calculated for 16 and 15 cells respectively from 4 independent experiments.

To study the effect on chromatin compaction of increasing cation concentrations in a more native environment, HeLa^H2B-2FP^ cells were permeabilised with 0.00125% digitonin for 7 minutes in a buffer containing 110 mM potassium acetate with 20 mM HEPES (pH 7.4). The cells were then washed in the same buffer lacking digitonin. Fluorescence lifetime measurements were acquired for a given set of cells in the presence of increasing concentrations of either Mg^2+^ or Ca^2+^. An increase in either Mg^2+^ or Ca^2+^ concentration leads to a decrease in the measured fluorescence lifetime of H2B-EGFP in HeLa^H2B-2FP^ cells, indicating an increase in chromatin compaction. The increase in compaction can also be visualised in the corresponding fluorescence intensity images (Fig. 5A). The change in levels of chromatin compaction were similar for increased concentrations of Mg^2+^and Ca^2+^ with a maximal decrease in H2B-EGFP lifetime at 6–8 mM of either Mg^2+^ or Ca^2+^(Fig. 5). These data are consistent with results obtained from *in vitro* experiments on nucleosome arrays, where maximal compaction was observed at 6–8 mM of Mg^2+^ [25]. As a control, excess EDTA was added to chelate free divalent cations and this showed that the fluorescence lifetime of EGFP in the HeLa^H2B-2FP^ cells increased again, indicating chromatin decompaction when the divalent cations are chelated (Fig. 5). However, the level of decompaction was not complete after addition of excess EDTA and EGFP fluorescence lifetime values correlated with those observed at a concentration of 2 mM of either divalent cation. We propose that EDTA is not able to fully sequester a subpopulation of divalent ions that are tightly bound to chromatin. Compaction of chromatin may result in more than one structural conformation, which may differ in stability. This could explain the rigidity of a structure formed at 2 mM of either Mg^2+^, or Ca^2+^, but reversible at higher ion concentrations, potentially through the formation of different higher order chromatin structures.

### Electron Spectroscopic Imaging of Permeabilised Cells with Increasing Concentration of Divalent Cations

**Figure 6:**
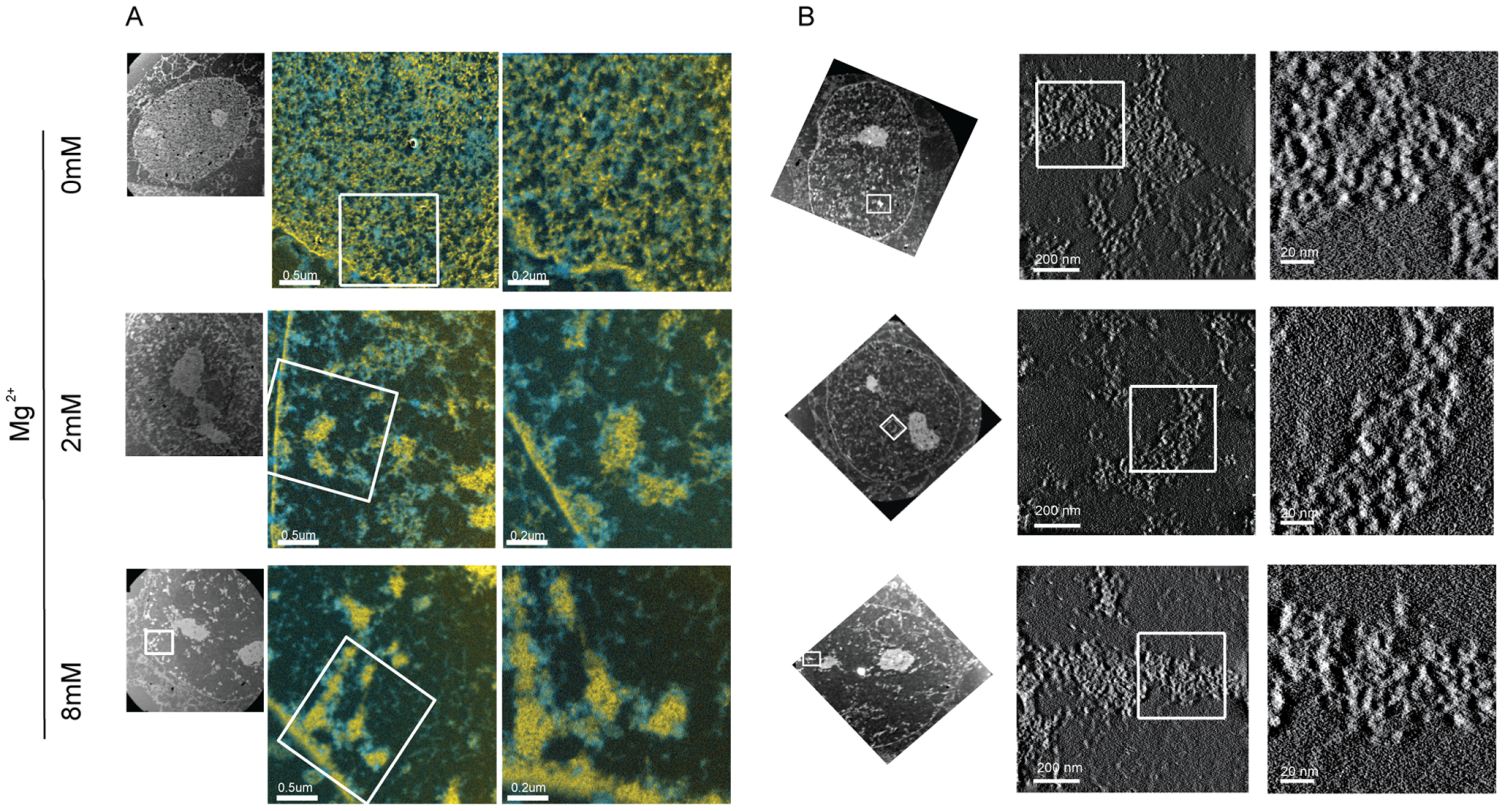
Electron spectroscopic imaging of permeabilised cells showing increased chromatin compaction with increase in Ca^2+^ concentration. (A) Electron spectroscopic imaging showing chromatin in yellow and non chromosomal proteins in blue of permeabilised HeLa cells containing varying levels of Mg^2+^ ions. (B) ESI combined with tomography showing the three dimensional structure of the chromatin of permeabilised HeLa cells containing varying levels of Mg^2+^ ions.

To analyse the chromatin compaction observed with the FLIM measurements at higher resolution, we used an independent technique, i.e., electron spectroscopic imaging (ESI) [26], to visualise chromatin in permeabilised cells, before and after addition of increasing concentrations of Mg^2+^. ESI provides quantitative, high-contrast images of chromatin fibres in situ with high spatial resolution, without the use of contrast agents [26]. Cells permeabilised in buffers containing either 0, 2 or 8 mM of exogenous magnesium ions were analysed using this technique. The ESI images show a drastic increase in chromatin compaction with increasing Mg^2+^ concentration. Chromatin in these permeabilised cells is diffuse at 0 mM Mg^2+^(Fig. 6). As the concentration of Mg^2+^ increases, the chromatin forms more condensed foci. At 8 mM of Mg^2+^ the chromatin condenses into foci, predominantly seen along the nuclear membrane and nucleolus.

When ESI is combined with electron tomography, the structural features of chromatin can be studied in higher detail. Notably, the ESI data do not show any evidence of 30 nm chromatin fibres in nuclei, even at the higher concentrations of Mg^2+^ (Fig. 6). This contrasts with studies on nucleosome arrays *in vitro*, which have shown the formation of 30 nm chromatin fibres at even the lower concentration of 2 mM Mg^2+^ [24].

### Relation of Intracellular Ca^2+^ to Chromatin Compaction

**Figure 7:**
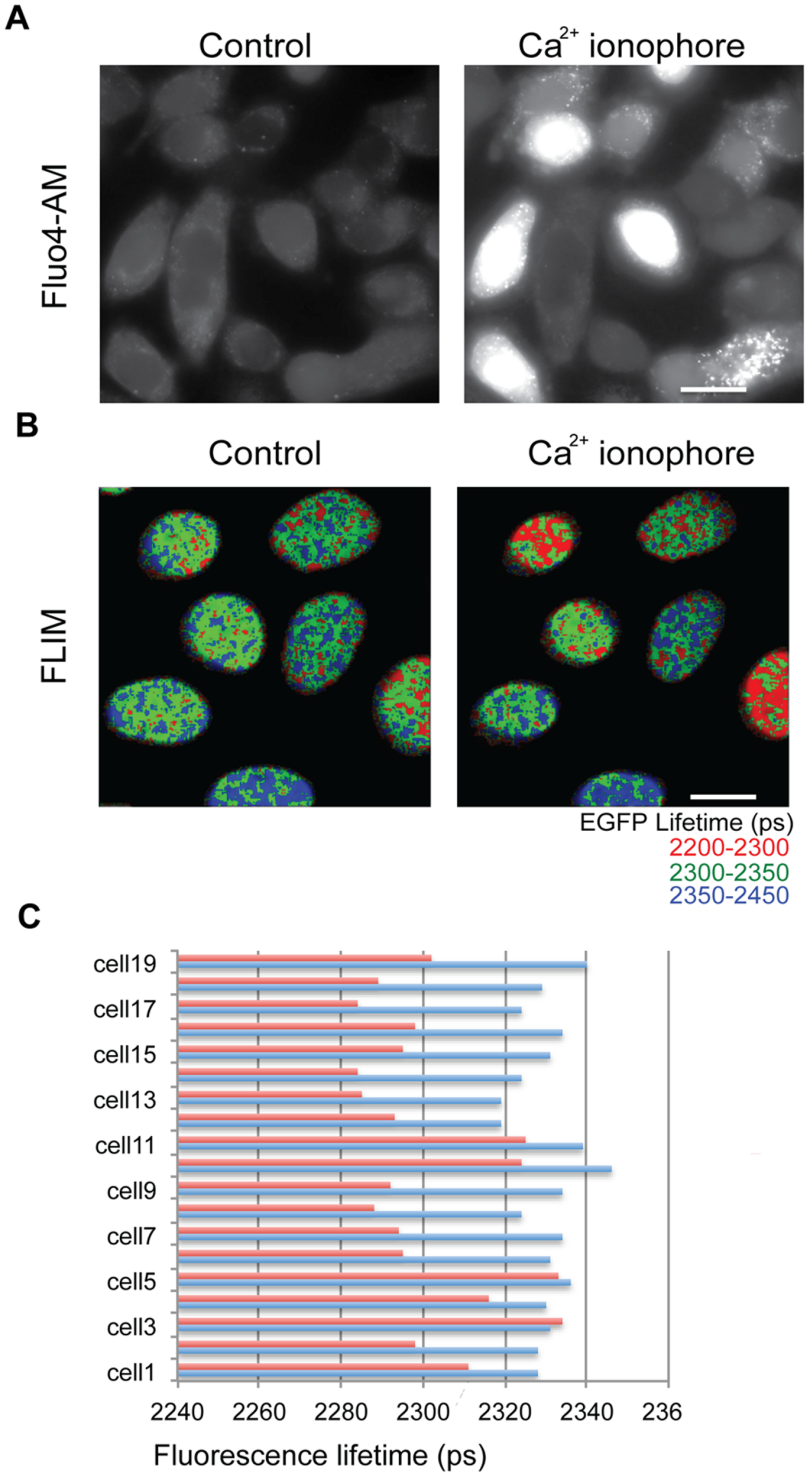
Increasing calcium concentration in live cells leads to an increase in chromatin compaction. (A) Fluo4 fluorescence images showing an increase in free intracellular calcium levels on treating the cells with Ca^2+^ ionophore. (B) FLIM images showing a decrease in fluorescence lifetime of Hela^H2B-2FP^ cells that have been treated with Ca^2+^ ionophore. (C) Chart showing the mean lifetime values of individual Hela^H2B-2FP^ cells before (blue) and after treating with Ca^2+^ ionophore. (Scale bar –10 µm).

The above experiments show that an increase in divalent cation concentration causes an increase in chromatin compaction in permeabilised cells. However, the chromatin in permeabilised cells, although it more closely resembles native chromatin when compared with defined nucleosome arrays assembled *in vitro*, may also not be identical to the chromatin structures formed in live cells. To test if chromatin condensation can be detected in live cells upon an *in vivo* increase in calcium, we treated cells with a Ca^2+^ ionophore (A23187) to increase the intracellular concentration of free calcium. To confirm that the addition of A23187 leads to an increase in intracellular calcium levels, HeLa cells were loaded with the dye fluo4-AM and imaged both prior to and after exposing cells to 10 µM A23187. Upon addition of A23187 there was an increase in fluorescence of fluo4, confirming the increase in intracellular Ca^2+^ (Fig. 7A).

To ascertain if this increase in calcium affects chromatin compaction, FLIM measurements on HeLa^H2B-2FP^ cells were acquired both before and after addition of A23187. A decrease in the measured EGFP fluorescence lifetime in cells was observed following the addition of the ionophore, indicating that the increased calcium levels correlated with increased compaction of chromatin (Fig. 7).

Next, we examined the relationship between ATP, Ca^2+^ levels and chromatin compaction. As demonstrated above, ATP depletion causes an increase in intracellular calcium concentration in the HeLa^H2B-2FP^ cells and high calcium levels correlate with an increase in chromatin compaction. Therefore, we examined whether the increased levels of calcium caused by ATP depletion are sufficient to explain the increase in chromatin condensation observed when ATP is depleted. To do this we took advantage of the fact that an increase in the levels of free intracellular calcium can be prevented using the cell permeable chelater, BAPTA-AM. When inside the cell BAPTA-AM is cleaved by esterases to form the active form – BAPTA, whereupon it loses its ability to traverse cellular membranes, thereby trapping it within cells [27].

**Figure 8:**
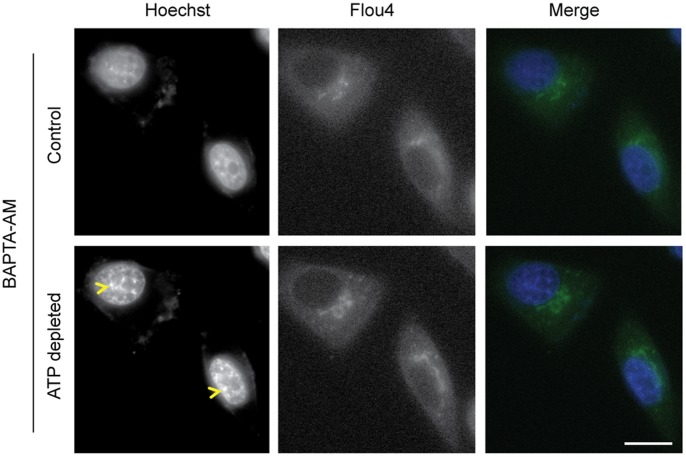
Preventing increase in free calcium after ATP depletion does not prevent chromatin compaction. Increase in free intracellular calcium upon ATP depletion can be prevented by preloading the cells with BAPTA, an intracellular Ca^2+^ chelater. Fluo4 fluorescence in HeLa cells preloaded with BAPTA does not change with ATP depletion, indicating no changes in the intracellular calcium concentration (p-value = 0.72). Increase in compaction of chromatin after ATP depletion (yellow arrow head) is seen from Hoechst staining. (Scale bar 10 µm).

HeLa cells were loaded with BAPTA-AM along with Fluo4-AM to image the changes in Ca^2+^. The HeLa cells were imaged both before and after depleting ATP with sodium azide and 2-deoxyglucose. In the presence of BAPTA, there was no increase in the fluorescence of Fluo4 (p value –0.72), showing that there was little or no increase in the level of free intracellular Ca^2+^ (Fig. 8). Interestingly, however, there was still an increase in the compaction of chromatin, as seen with Hoechst 33342 staining (Fig. 8). Therefore, preventing the increase in free intracellular calcium that accompanies ATP depletion, does not prevent chromatin compaction. We conclude that an alternative mechanism must contribute to promoting chromatin compaction when ATP is depleted.

### Chromatin Compaction in Permeabilised Cells Increases with Increasing Polyamine Concentration

**Figure 9:**
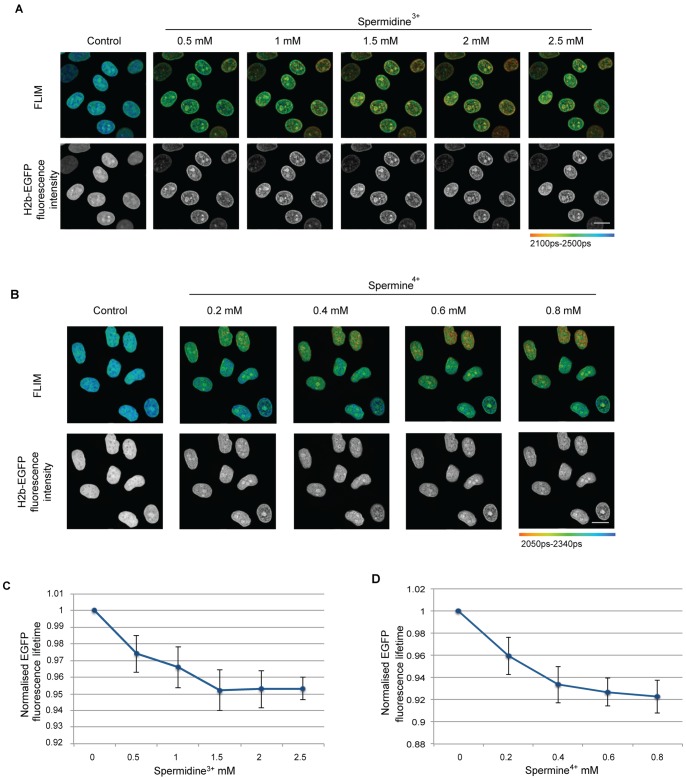
Increase in polyamine concentration leads to increased chromatin compaction in permeabilised cells. (A) FLIM images of permeabilised Hela^H2B-2FP^ cells with increasing Spermidine^3+^ concentration showing a reduction in EGFP fluorescence lifetime. The increase in compaction is also seen with the fluorescence intensity image. (Scale bar –10 µm) (B) FLIM images of permeabilised Hela^H2B-2FP^ cells with increasing Spermine^4+^ concentration showing a reduction in the calculated EGFP fluorescence lifetime. The increase in compaction is also seen with the fluorescence intensity image. (Scale bar –10 µm) (C) The average fluorescence lifetime normalised to control values of the Hela^H2B-2FP^ cells are plotted against the Spermidine^3+^ concentration. Error bars indicate standard deviation for values calculated for 15 cells from 3 independent experiments. (D) The average fluorescence lifetime normalised to control values for each the cells are plotted against the Spermine^4+^ concentration. Error bars indicate standard deviation for values calculated for 22 cells from 3 independent experiments.

We next examined whether increasing concentrations of the polyamines, spermine^+4^ and spermidine^+3^, cause an increase in chromatin compaction in permeabilised HeLa^H2B-2FP^ cells. The concentration of polyamines was gradually increased and FLIM measurements were acquired, showing that increasing the levels of polyamines resulted in an increase in chromatin compaction (Fig. 9). There was an inverse correlation between the charge of the polyamine and the concentration required for a given degree of compaction. Maximum compaction was seen at 1.5 mM of spermidine and at 0.4 mM of spermine, as judged both by changes in the EGFP lifetime and by visual inspection of DAPI stained images. A similar level of compaction to that brought about by addition to the permeabilised cells of 6–8 mM divalent cations arises at either 1.5 mM of spermidine or at 0.2–0.4 mM of spermine. Thus, these data are consistent with the hypothesis that small changes in the localization of polyamines can potentially cause major changes in the levels of chromatin compaction.

### Chromatin Compacts in Permeabilised Cells upon RNase Treatment

**Figure 10:**
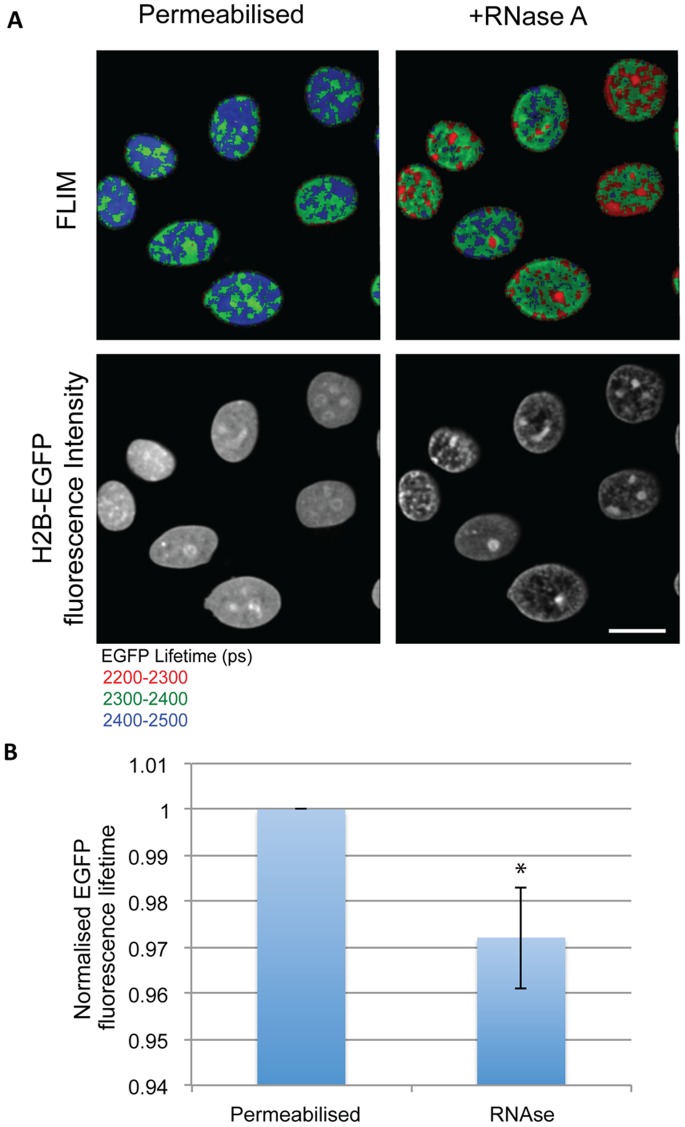
Chromatin of permeabilised cells compacts with the addition of RNase A. (A) FLIM images show the decrease in EGFP fluorescence lifetime in permeabilised Hela^H2B-2FP^ cells that have been treated with RNase A. The increase in compaction is also seen in the intensity images, which show fluorescence from H2b-GFP. (Scale bar –10 µm) (B) The chart shows the average decrease in EGFP fluorescence lifetime normalised to the values of control cells. Error bars indicate standard deviation for values calculated for 13 cells from 3 independent experiments. (* p-value = 9.89×10^−7^).

Under normal cell growth conditions significant levels of RNA associate with chromatin. The presence of RNA can increase the overall negative charge of chromatin. RNA can also compete with DNA to complex with positively charged molecules that are able to reduce the net negative charge of chromatin. If the hypothesis is correct that the conformation and compaction state of native chromatin is strongly charge dependent, then removal of the negatively charged RNA from chromatin should also result in an increase in compaction. To test this we therefore treated permeabilised HeLa^H2B-2FP^ cells with RNase A and then acquired FLIM images. This resulted in an increase in compaction of chromatin, as judged both from the resulting decrease in H2B-EGFP fluorescence lifetime and by the change in the H2B-EGFP fluorescence intensity images (Fig. 10). These data support the hypothesis that charge repulsion effects may play a significant role in determining the extent of chromatin compaction in cell nuclei.

## Discussion

In this study we have used a combination of high resolution fluorescence and electron microscopy techniques to analyse the higher order organisation and compaction of chromatin within the nuclei of human cells. Specifically, we have analysed the reversible hyper compaction of chromatin that results when ATP levels are depleted by treating cells with sodium azide and 2-deoxyglucose. We show this ATP-dependent chromatin compaction is not caused specifically by loss of transcription activity and that it occurs both in interphase and mitotic cells. Using a previously described FLIM-FRET assay for measuring chromatin compaction in intact live cells, we show using both live and permeabilised cells that chromatin conformation within nuclei is strongly influenced by the levels of divalent cations. However, while we observe that ATP depletion results in an increase in the level of unbound calcium within cells, chromatin condensation still occurs even in the presence of a calcium chelator. We also show that chromatin compaction is strongly affected by small changes in the levels of polyamines, which are known to bind ATP. The data show that the higher order structure of chromatin within nuclei can be rapidly and reversibly altered *in vivo* by changes in the levels of free cations and polyamines.

We observed here that the rapid compaction of chromatin induced by ATP depletion was not confined to chromosomes in interphase and also took place in mitotic cells. There are several implications of this result regarding the possible mechanism causing chromatin condensation when ATP is depleted. First, as mitotic chromosomes do not replicate and show little or no ongoing transcription, it excludes the explanation that hyper-condensation is the indirect consequence of interfering with either transcription, or DNA replication, i.e., major ATP-dependent nuclear processes that will be halted when ATP levels are depleted. Consistent with this inference, we showed that directly blocking transcription in interphase cells with chemical inhibitors also did not cause the level of chromatin compaction evident when ATP levels are depleted. Thus, even though transcription has been implicated in maintaining decompacted chromatin and ATP depletion will result in the inhibition of transcription [28], [29], other mechanisms must also affect chromatin conformation upon ATP depletion. Second, the observations in mitotic cells indicate that the structural transition that causes hyper-compaction of chromatin when ATP is depleted is likely distinct from the structural change that compacts interphase chromatin into the more condensed state evident when cells enter mitosis. Further, it indicates that mitotic chromosomes, despite being already more compacted than interphase chromosomes, are nonetheless capable of undergoing further structural transitions to a still more compacted state. Consistent with this observation, in a recent study we have also shown that down regulation of the Adenamatosis polyposis coli (APC) protein results in a higher level of condensation of mitotic chromosomes [30]. Therefore, we conclude that ATP depletion probably does not trigger condensation specifically by activating the mechanisms that cause chromatin to compact in mitosis.

Studies with nucleosome arrays *in vitro* have shown that chromatin condenses with increasing concentrations of polyvalent cations, in a charge and concentration dependent manner [20]. The data indicate that charge neutralisation of the sugar phosphate backbone plays a role in maintaining chromatin compaction. An increase in calcium on mitotic chromosomes has also been observed [2]. We show here that ATP depletion in intact, live cells leads to a clear increase in the level of free intracellular calcium that can be visualised using a calcium -binding fluorophore. Further experiments using permeabilised HeLa^H2B-2FP^ cells showed that increasing levels of either of the divalent cations Ca^2+^ or Mg^2+^ caused a near identical increase in chromatin compaction. We suggest that this cation-dependent *in vivo* compaction likely arises mainly from charge neutralisation of the sugar phosphate backbone, as previously reported for *in vitro* studies of cell free nucleosome arrays. This is supported by our observation that the increase in compaction was cation concentration dependent and saturated at about 6–8 mM of either of the divalent ions, similar to that reported for nucleosome arrays *in vitro* [25], [31].

Consistent with chromatin compaction arising via a charge neutralisation mechanism, subsequent chelation of the divalent cations by addition of 12 mM EDTA to the permeabilised HeLa^H2B-2FP^ cells that had been incubated with 10 mM of divalent cations resulted predominantly in a reversal of compaction. However, the reversal of compaction observed upon adding excess EDTA was not complete but decreased to a similar compaction level, as judged by the FLIM-FRET signal, to that seen in the permeabilised cells treated with 2 mM of divalent cations. We suggest two possibilities that could explain this effect. First, the chromatin may have multiple sites for binding to the cations, which differ in their binding affinity, and a subset of these sites have a binding affinity higher than that of EDTA. Second, the chromatin may form two or more distinct forms of higher order compacted structures, some of which are more stable and/or more resistant to reversal within the conditions of the permeabilised cell system.

We considered it likely that one of these higher ordered structures discussed above might correspond to the 30 nm chromatin fibre that has been extensively described using nucleosome arrays reconstituted under cell-free, *in vitro* conditions [24], [32], [33], [34]. This 30 nm fibre is promoted by incubation with increasing levels of divalent cations and can be detected forming *in vitro* already with 2 mM MgCl_2_ [24]. However, it has been difficult to confirm that chromatin indeed forms 30 nm fibres *in vivo* in intact mammalian cells. Although 30 nm fibres have been reported in starfish sperm and in permeabilised avian erythrocyte nuclei [35], [36], currently there is no evidence for the presence of the 30 nm fibre in intact mammalian cells [37]. In this regard it is interesting that we also failed to detect any 30 nm fibres in HeLa cell nuclei, even using very high resolution electron spectroscopic imaging (ESI) [26]. While we could visualise by ESI a major increase in the level of chromatin compaction in permeabilised nuclei upon incubation with increasing levels of magnesium ions, corresponding to divalent cation concentrations that promote 30 nm fibre formation *in vitro*, none of the ESI images of human cell nuclei showed structures corresponding to 30 nm fibres. This was a surprising result, given the similarity in magnesium-dependent compaction dynamics observed between our current data using intact nuclei and previous studies on cell free nucleosome arrays *in vitro*. Therefore, this raises the question as to whether either the 30 nm chromatin fibre that forms *in vitro*, or some other forms of higher order structures, are most relevant to the organization of chromatin within the complex environment of mammalian cell nuclei.

Despite the ability of divalent cations to promote chromatin condensation in permeabilised cells, and our observations that ATP depletion result in increased levels of free calcium, it is not clear that changes in divalent cation levels alone are responsible for the compaction of chromatin when ATP is depleted. In particular, we observed that chromatin still compacts when ATP is depleted, even when a calcium chelater is present to bind to the free calcium released. This led us to look at the possible involvement of other polyvalent cations. Polyamines are naturally occurring positively charged small molecules. They have been shown to be essential for growth [38]. The intracellular levels of polyamines are regulated, with decreased levels leading to a reduction of nucleic acid and protein synthesis, eventually causing cell growth inhibition [39]. The concentrations of polyamines *in vivo* are also highly regulated in a cell cycle dependent manner [40]. Polyamines are positively charged in solution at physiological pH, ranging from +2 to +4. Furthermore, it has been shown that the addition of polyamines to nucleosome arrays *in vitro* causes compaction, as judged by the increase in their sedimentation coefficient [20].

Several lines of evidence support the possibility that polyamines such as spermine and spermidine can affect chromatin conformation *in vivo* and may contribute to promoting the reversible hyper-compaction of chromatin seen upon ATP depletion/repletion. Previous studies have shown that spermine has a higher affinity towards ATP than DNA, while spermidine has its highest affinity towards ATP, followed by RNA and DNA [41]. Thus, after ATP depletion, it is expected that there will be an increase in the pool of spermine and spermidine, which was previously bound to ATP, that is now able to bind DNA (and RNA), thereby neutralising the negative charge on the polyphosphate backbone and effecting chromatin compaction. This is supported by preliminary immunostaining data using an anti-spermine antibody, showing that the staining pattern markedly changes following depletion of ATP with sodium azide and 2-deoxyglucose (our unpublished observations). We observed using the permeabilised HeLa^H2B-2FP^ cells that when the concentration of either of the polyamines spermine or spermidine was increased, there was a corresponding increase in the compaction of chromatin. As judged using the FLIM-FRET assay, the increase in compaction was maximal and saturated at about 1.5 mM spermidine (+3) and 0.4 mM spermine (+4). The fact that lower concentrations of the more highly charged polyamines were needed to promote maximal compaction, as compared with the 6–8 mM of divalent cations (+2), is consistent with the mechanism of compaction involving charge neutralisation of the sugar phosphate DNA backbone.

We note that the concentration of polyamines has been reported to increase in cells that have mutations in the Adenamatosis polyposis coli (APC) gene [42]. The role we hypothesise here for polyamines in promoting chromatin compaction *in vivo* is thus consistent with our recent report using the FLIM-FRET assay that there is an increase in the compaction of mitotic chromosomes in cells deficient for APC, when compared with wild type cells [30]. Depletion of ATP also prevents transcription and therefore will result in a decrease in nascent RNA levels in the nucleus. One consequence of reduced RNA levels will be to remove another reservoir of polyamine binding sites and thus potentially increase the pool of polyamines able to bind DNA. Consistent with this idea, it has been reported previously that chromatin compaction increases when RNA levels are reduced [43]. We obtained similar results in this study using the FLIM-FRET assay in HeLa^H2B-2FP^ cells. Thus, we observed that removal of bulk RNA by RNase A treatment resulted in an increase in chromatin compaction. Similar evidence to support the hypothesis that RNA can facilitate the maintenance of an open chromatin structure has been recently studied by Schubert et.al., [44]. In their study they describe an RNA dependent mechanism to maintain open chromatin structure. The study describes a protein Df31 in Drosophila, which localises to euchromatin and is also bound to RNA. These data therefore support a role for RNA in maintaining decompacted chromatin. Given our present data we suggest that this role of RNA may be connected, at least in part, with modulating the charge environment in the nucleus via binding to cations.

In summary, this analysis of chromatin conformation in HeLa cell nuclei has provided evidence indicating that charge neutralisation of the sugar-phosphate DNA backbone by divalent cations and polyamines can strongly influence the higher order structure and compaction of chromatin *in vivo*. The data are consistent with a model in which the increase in the intracellular pool of polyamines and divalent cations resulting from depletion of ATP contributes to the large scale hyper-compaction of chromatin that is seen to occur *in vivo* when ATP is depleted by sodium azide and 2-deoxyglucose. In this model the rapid and reversible chromatin hyper-compaction is mediated, at least in part, through the equilibration of polyamines and divalent cations binding either to ATP and RNA, or else to DNA, where it can neutralise the negative charge on the DNA backbone and hence promote compaction.

Nonetheless, multiple ATP-dependent enzymes, including helicases, chromatin remodeling factors and kinases are also known to play important roles in modulating chromatin conformation in many situations. None of our present data exclude that the loss of function of one or more such activities may also contribute to the reversible compaction of chromatin observed when ATP is depleted, either during interphase, or mitosis, or both. Given that many of these ATP-dependent enzymes can play redundant roles in the cell, it is difficult to definitely conclude whether they are involved, even if multiple parallel knock-out experiments are involved. It will however be interesting in future to examine this further and to assess whether the regulation of polyamine availability can contribute towards physiological mechanisms for controlling higher order chromatin structure *in vivo*.

## Materials and Methods

### Cell Culture

HeLa cells were cultured in Dulbecco’s Modified Eagles’ Medium (DMEM) (Invitrogen) supplemented with 10% FBS, 100 U/ml penicillin-streptomycin in a humidified incubator at 37°C with 5% CO_2_. HeLa^H2B-2FP^, stably expressing EGFP and m-Cherry tagged histone H2B [11] were cultured in DMEM medium supplemented with 10% FBS, 100 µg/ml penicillin-streptomycin and containing 2 µg/ml blasticidin-S and 200 µg/ml of G418.

### Fluorescence Lifetime Measurements by Time-Correlated Single-Photon Counting (TCSPC)

Förster or Fluorescence Resonance Energy Transfer (FRET) and molecular crowding can be used to image direct protein-protein interactions in cells. While transferring energy from an excited donor to an acceptor [45], FRET decreases the donor fluorescence and increases the acceptor fluorescence. Because the energy transfer is highly distance-dependent, detection of FRET requires that the two fluorophores must be within ∼1 to 10 nm, i.e., the distance typically found for directly interacting proteins [46]. In this study, as a quantitative readout of FRET, we measured the fluorescence lifetime of the donor, defined as the average time between fluorophore excitation and photon emission by using Fluorescence Lifetime Imaging Microscopy (FLIM).

Fluorescence lifetime Imaging Microscopy (FLIM) was performed using an inverted laser scanning multiphoton microscope Radiance 2100MP (Bio-Rad Laboratories) with a 60x oil immersion (1.4 NA). The microscope is equipped with an environmental chamber suitable to maintain the live cells and optics at constant temperature (37°C). The chamber is constructed with black walls to exclude external sources of light during the sensitive period of FLIM measurement. Two-photons excitation was achieved using a Chameleon Verdi-pumped ultrafast tunable (720–930 nm) laser (Coherent) to pump a mode-locked frequency-doubled Ti:Sapphire laser that provided sub-200-femtosecondpulses at a 90-Mhz repetition rate with an output power of 1.4 W at the peak of the tuning curve (800 nm). Enhanced detection of the scattered component of the emitted (fluorescence) photons was afforded by the use of fast single-photon response (5783P; Hamamatsu Photonics) direct detectors.

The fluorescence lifetime imaging capability was provided by TCSPC electronics (SPC- 830; Becker & Hickl GmbH).

EGFP/mCherry was used as a FRET pair for all the FLIM measurements. The optimal two-photon excitation wavelength to excite the donor (EGFP) was determined to be 890 nm. Fluorescence emission of EGFP fusion proteins was collected using a bandpass filter (528±25 nm) to limit detection to only the donor fluorophore (EGFP) and prevent contamination from the acceptor (mCherry) emission [12]. Laser power was adjusted to give a mean photon count rate of the order 10^4^–10^5^ photons/s. Fluorescence lifetime measurements were acquired over 60 s. Fluorescence lifetimes were calculated for all pixels in the field of view (256×256 pixels) or for a particular selected region of interest (e.g., nucleus) using SPCImage software (Becker & Hickl, GmbH).

### Drug Treatments

Cells were grown in petri dishes to about 70% confluency. The cells were treated with various drugs to cause effects as listed below.

### ATP Depletion

The cells were treated with a final concentration of 10 mM sodium azide (Sigma-Aldrich ♯S2002) and 50 mM 2-deoxy glucose (Sigma-Aldrich ♯D8375), which causes the depletion of ATP in cells by inhibiting the oxidative and glycolytic pathways simultaneously [47]. The chemicals were either dissolved directly in imaging media or a 10X stock was made with PBS and added to media.

### Calcium Ionophore (A23187)

The cells were treated at a final concentration of 10 µM of calcium ionophore (Sigma-Aldrich ♯C7522) from a stock solution of 2 mM in DMSO stored at -20°C. The addition causes an increase in concentration of free intracellular calcium.

### Transcription Inhibition

For inhibition of transcription, cells were treated with 5,6-Dichloro-1-beta-D-ribofuranosylbenzimidazole (DRB) at a final concentration of 25 µg/ml for 1 hour. Inhibition of transcription was visualised by staining nascent RNA using Click-iT® RNA Alexa Fluor® 488 Imaging Kit (Invitrogen ♯C10329) following the manufacturer’s instructions.

### Cell Permeabilisation

Cell permeabilisation was carried out on cells grown to ∼70% confluency in glass-bottomed petri dishes (Willcowells ♯GWSt-3522). The cells were washed once with PBS and incubated in permeabilisation buffer (20 mM HEPES pH-7.4, 110 mM Potassium acetate, 0.00125% digitonin) for 7 minutes at room temperature. The cells were then washed in the permeabilisation buffer lacking digitonin for 3 minutes, washes were repeated three times. Further treatments were carried out with addition of cations or RNase A in permeabilisation buffer lacking digitonin.

### RNase A Treatment

Cells treated with RNase A were first permeabilised using the above-mentioned protocol. RNase A was then added at a final concentration of 10 µg/ml.

### Ca^2+^ ion Measurements

Calcium measurements were preformed using the cell permeable calcium specific dye, Fluo4-AM (Invitrogen ♯F-14201). The cells were grown to about 70% confluency. The media was removed and the cells were washed once with serum free DMEM (Invitrogen ♯41966-052). 1 µl of 1 mM Fluo4-AM in DMSO was mixed with 1 µl of 20% Pluronic F-127 (Invitrogen ♯P6867) and added to 1 ml of serum free DMEM which was then added to the cells and incubated for 40 minutes at 37°C. For experiments containing BAPTA-AM, 2 µl of 25 mM BAPTA-AM in DMSO was added to the Fluo4-AM, Pluronic F-127 mixture prior to the addition of serum free DMEM. Pluronic F-127 is a surfactant that helps loading the dye into the cells. The cells were then washed once with CO_2_ independent medium lacking phenol red (Invitrogen ♯ME090085P1) and imaged with the same media. Fluo4 was imaged with excitation at 488 nm and emission at 525 nm in a 37°C chamber attached to the microscope. All images were acquired with a wide-field fluorescence microscope (DeltaVision Spectris; Applied Precision) and a CoolMax charge-coupled device camera (Roper Scientific). Imaging was performed using a 60x oil immersion, NA 1.4 Plan-Apochromat objective. Quantification of change in Fluo4 fluorescence was carried out using the ImageJ software, with a region of interest drawn within the nuclear regions of cells and mean arbitrary fluorescence units compared.

### ESI Microscopy

Sample preparation and detailed ESI procedure are described fully in Ahmed et al., (2009). HeLa cells were fixed in 1% glutaraldehyde, dehydrated and embedded in Quetol (Electron Microscopy Sciences). Samples were sectioned by an ultramicrotome (Leica) 70 nm. Energy filtered electron micrographs were taken on a transmission electron microscope (Tecnai 20, FEI) operated at 200 kV collected using a GATAN post column imaging filter at 120 and 155, and 385 and 415 eV to generate the phosphorus and nitrogen images, respectively. Pre- and post-edge images were recorded on a CCD camera. Digital micrograph software was used to collect images. Nitrogen images were subtracted from phosphorus images, so that the net nitrogen signal in chromatin structures was normalized to zero. These phosphorus subtracted nitrogen images are coloured blue. The phosphorus images are pseudo-coloured yellow and overlaid onto the phosphorus subtracted nitrogen image. Images were processed with Digital micrograph and Photoshop 7.0 (Adobe).

### Electron Tomography

Phosphorus ESI jump ratio tilt series of chromatin of sectioned HeLa cells were acquired at 120 and 155 eV using SerialEM [48] with 2° increment over a tilt range of ±60°. The images in the series were aligned and processed into jump ratios using a combination of IMOD [49] SPARX [50] and ImageJ. Three-dimensional phosphorus maps were reconstructed using the IMOD implementation of SIRT from the aligned jump ratio tilt series.

### Statistics

Statistical significance (p value) was calculated by comparing the indicated sample sets with their corresponding control values in Microsoft excel using a paired 2 tailed t test.
